# First-Degree Heart Block: The Guiding Light to Discovering an Aortic Root Abscess

**DOI:** 10.7759/cureus.12159

**Published:** 2020-12-18

**Authors:** Mitra Patel, Connor Grotton, Sreeram Ravi, Sarah Benson, Ronak G Soni

**Affiliations:** 1 Medicine, University of Toledo College of Medicine, Toledo, USA; 2 Cardiovascular Medicine, University of Toledo College of Medicine, Toledo, USA

**Keywords:** infectious endocarditis, perivalvular abscess, complete heart block, first degree heart block, pr prolongation

## Abstract

Minor conduction abnormalities such as first-degree heart blocks are generally overlooked on electrocardiogram (EKG) as their impact on clinical management is usually not substantial. However, they can be an important screening tool for early diagnosis of infective endocarditis (IE) and associated perivalvular complications, especially in patients with surgical valve replacements.

This case report describes a 58-year-old male with a past medical history of bicuspid aortic valve status post replacement five years prior to presentation who initially presented with presumed symptoms of a complicated urinary tract infection (UTI) and later developed chest pain and shortness of breath. He showed no initial signs of infection including negative blood and urine cultures. EKG showed new onset prolonged PR interval. He then underwent a transthoracic echocardiogram (TTE) which showed prosthetic valve dysfunction and subsequently underwent transesophageal echocardiogram (TEE) which revealed vegetations on all leaflets and circumferential peri-aortic abscess encompassing both coronary ostia and extending towards the tricuspid and mitral valve leaflets. The patient then underwent redo-sternotomy for dissection of mediastinal adhesions, extraction of the aortic bio-prosthesis, and debridement of the aortic root abscess. The aortic root was replaced with a homograft and the valve cultures were positive for *Enterococcus faecium*. The patient developed complete heart block afterwards and received a permanent pacemaker; repeat cultures showed no further evidence of infection.

This case report is presented to reiterate the importance of early detection of IE-related aortic valve abscess and their rare sequelae. Early screening for conduction abnormalities via EKG and subsequently a TEE can allow prompt identification and management of valvular abnormalities to prevent life-threatening complications and improve patient outcomes.

## Introduction

Infective endocarditis (IE) is an infection of the lining of the endocardium of the heart. This condition is uncommon in physiologically normal hearts, but the risk is increased in hearts with some form of abnormality such as congenital defects, damaged valves, or valve replacements [1]. The incidence of IE is about 15 per 100,000 people and has been increasing as the diagnostic criteria become clearer [2]. The people at the highest risk of developing IE are those with artificial valves, heart defects, or a history of IV drug use [3]. In these populations, formation of vegetations and subsequent abscesses are relatively high, which can cause life-threatening causes in even young patients.

Of the roughly 10,000 new cases of IE annually, some develop a perivalvular abscess with the aortic valve most commonly involved and the mitral valve the next most commonly involved. The incidence of perivalvular abscess varies significantly but the grim prognosis without treatment does not. Modalities of treatment include prompt medical management with antibiotics and surgical resection, which are necessary to have a favorable outcome [4-6]. The significance of a perivalvular abscess is that the mass effect of a growing abscess can prolong the PR interval leading to conduction abnormalities and eventual complete heart block without early recognition, which is why it is imperative to promptly recognize these conditions to have a favorable prognosis [7]. The latency from initiation of abscess formation to treatment has been shown to play an integral role in mortality of IE patients presenting with new PR interval prolongation [8]. Transthoracic echocardiogram (TTE) and transesophageal echocardiogram (TEE) have been proven to be able to detect the majority of potentially fatal abscesses around native and prosthetic valves and are important modalities to prevent fatal complications in these patients [9].

## Case presentation

**Figure 1:**
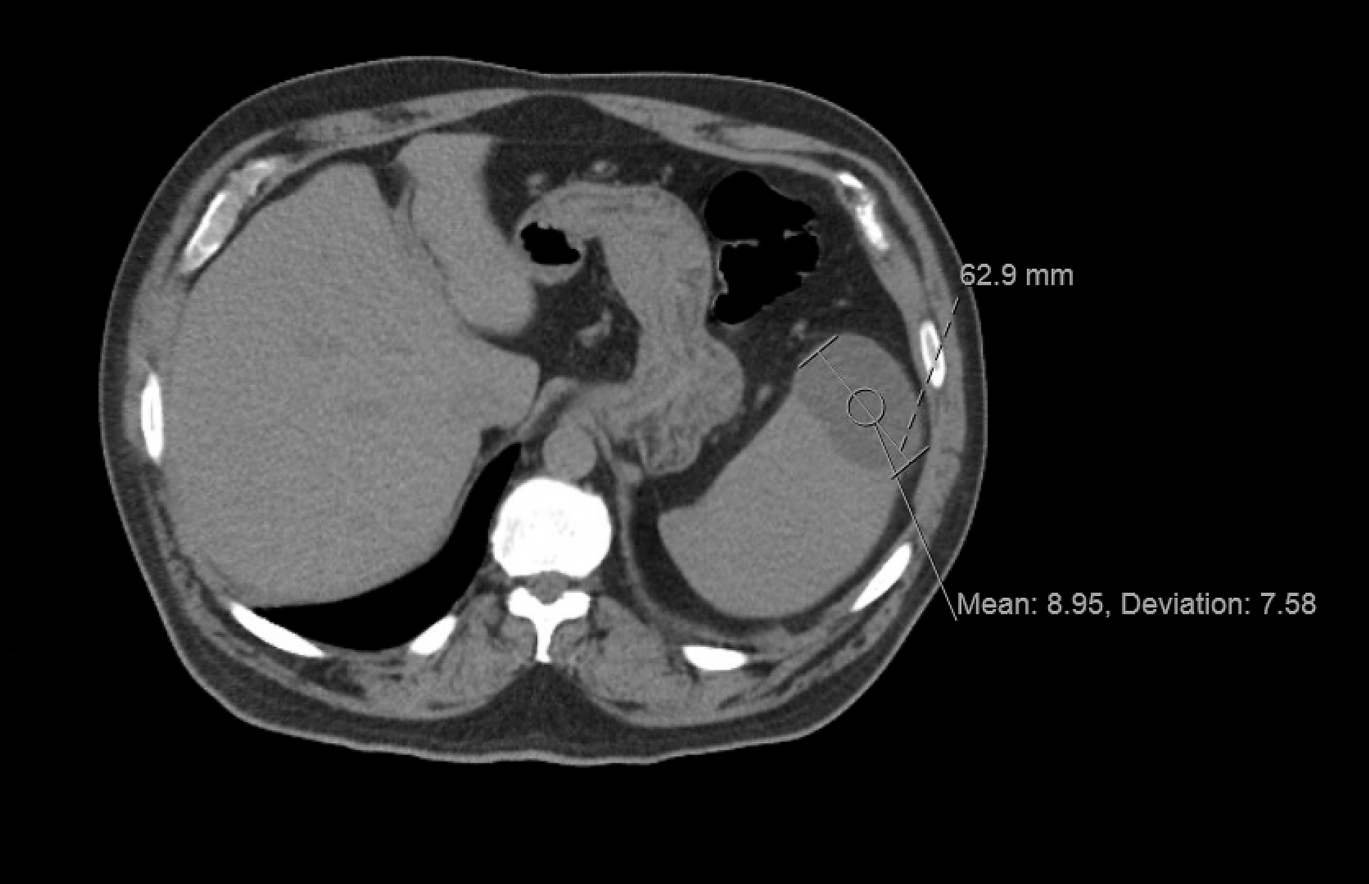
Splenic lesion due to suspected infection vs. infarction.

A 58-year-old male presented to the hospital with a two-day history of pressurized, substernal chest pain along with shortness of breath. These symptoms had been occurring intermittently for the past three months. The patient reported one particular episode of shortness of breath and dizziness upon standing three months earlier which led to a fall down the stairs. He had a history of a bicuspid aortic valve that was replaced with a bioprosthetic valve five years prior to admission. Two weeks earlier, the patient was prescribed oral trimethoprim-sulfamethoxazole for a presumed urinary tract infection (UTI), but afterwards he developed dyspnea, chest pain, and left flank pain. The patient underwent a CT scan one week prior to his presentation today that showed splenomegaly with three lesions, the largest of which measured 6.3 cm by 4.3 cm in diameter (Figure 1).

**Figure 2:**
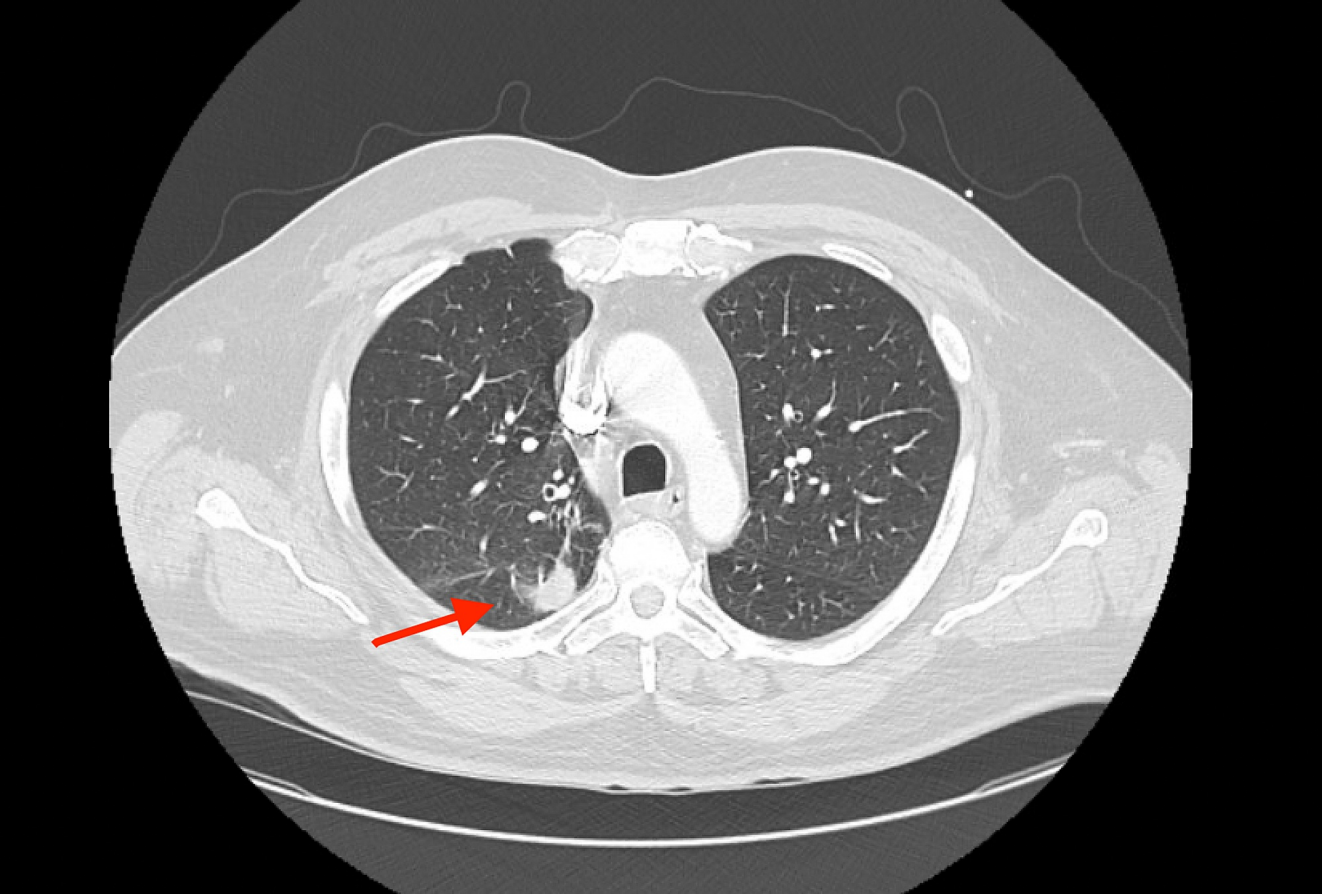
CT of the chest without significant findings, besides a small, linear right upper lobe infiltrate (red arrow).

In the ED, he was afebrile and hemodynamically stable. The remainder of his physical exam including cardiac exam was unremarkable. An electrocardiogram (EKG) was performed and showed a chronic right bundle branch block as well as a new first-degree heart block with a PR interval of 320 ms. A CT of the chest (CTA) and TTE were performed. The CT revealed right upper lobe linear infiltration suspicious for infection but no suspicion for pulmonary emboli (PE) (Figure 2) and the TTE showed prosthetic valve dysfunction.

**Figure 3:**
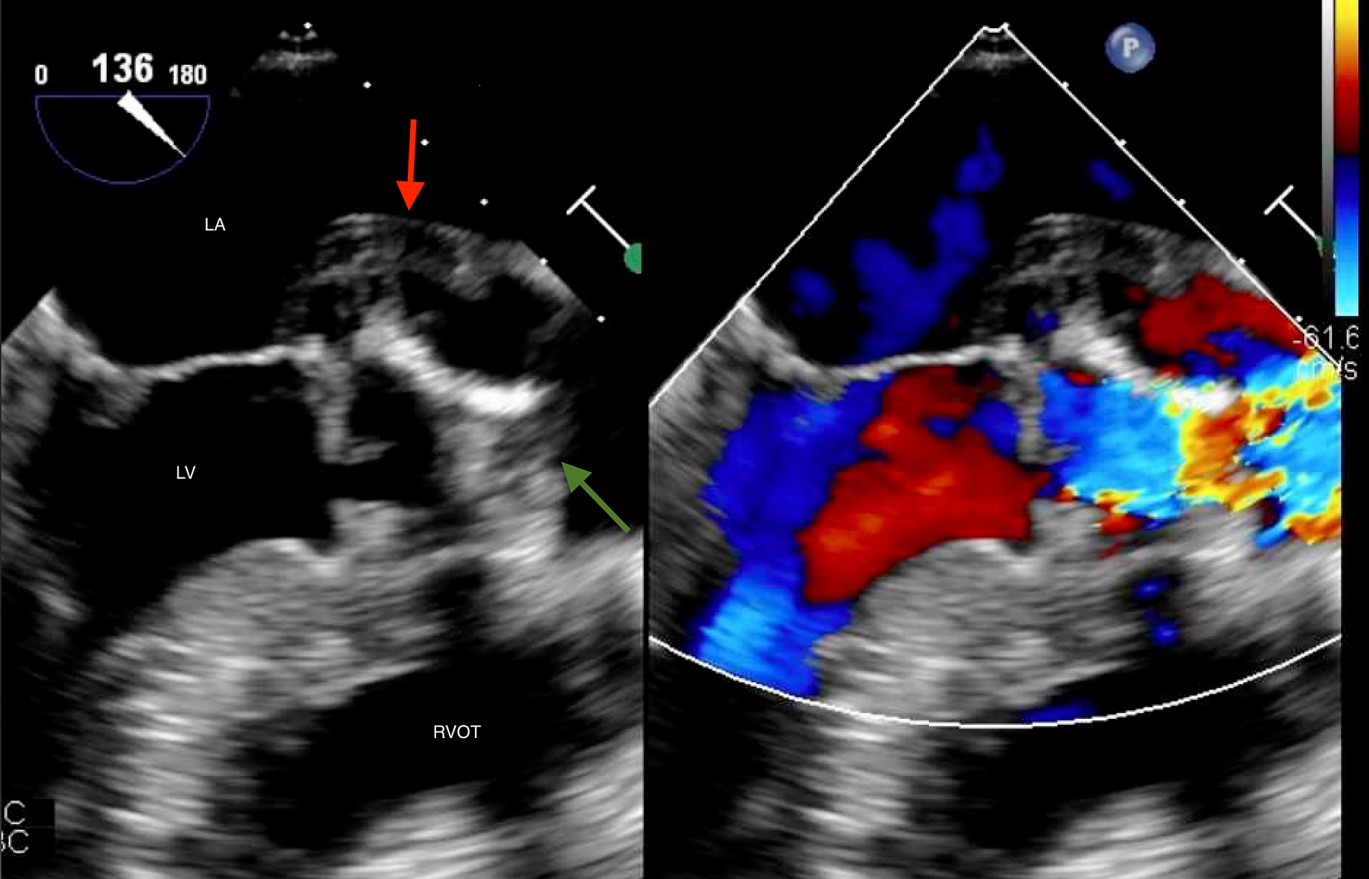
Midesophageal long axis view on TEE shows (green arrow) a vegetation on the aortic valve which leads to significant aortic regurgitation (red arrow). The lucency is highly suspicious for aortic valve abscess formation. TEE, transesophageal echocardiogram

**Figure 4:**
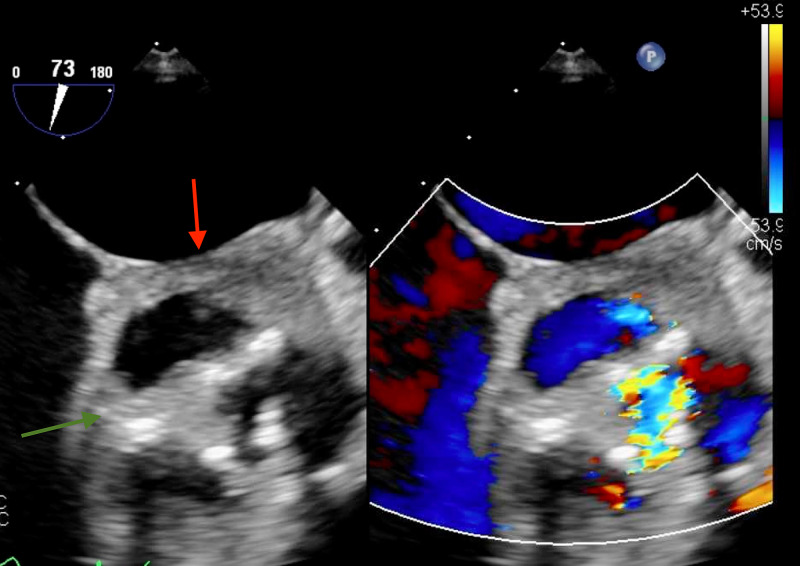
TEE of the aortic valve showing vegetations (green arrow) along with surrounding lucency suggestive of abscess formation (red arrow). TEE, transesophageal echocardiogram

**Figure 5:**
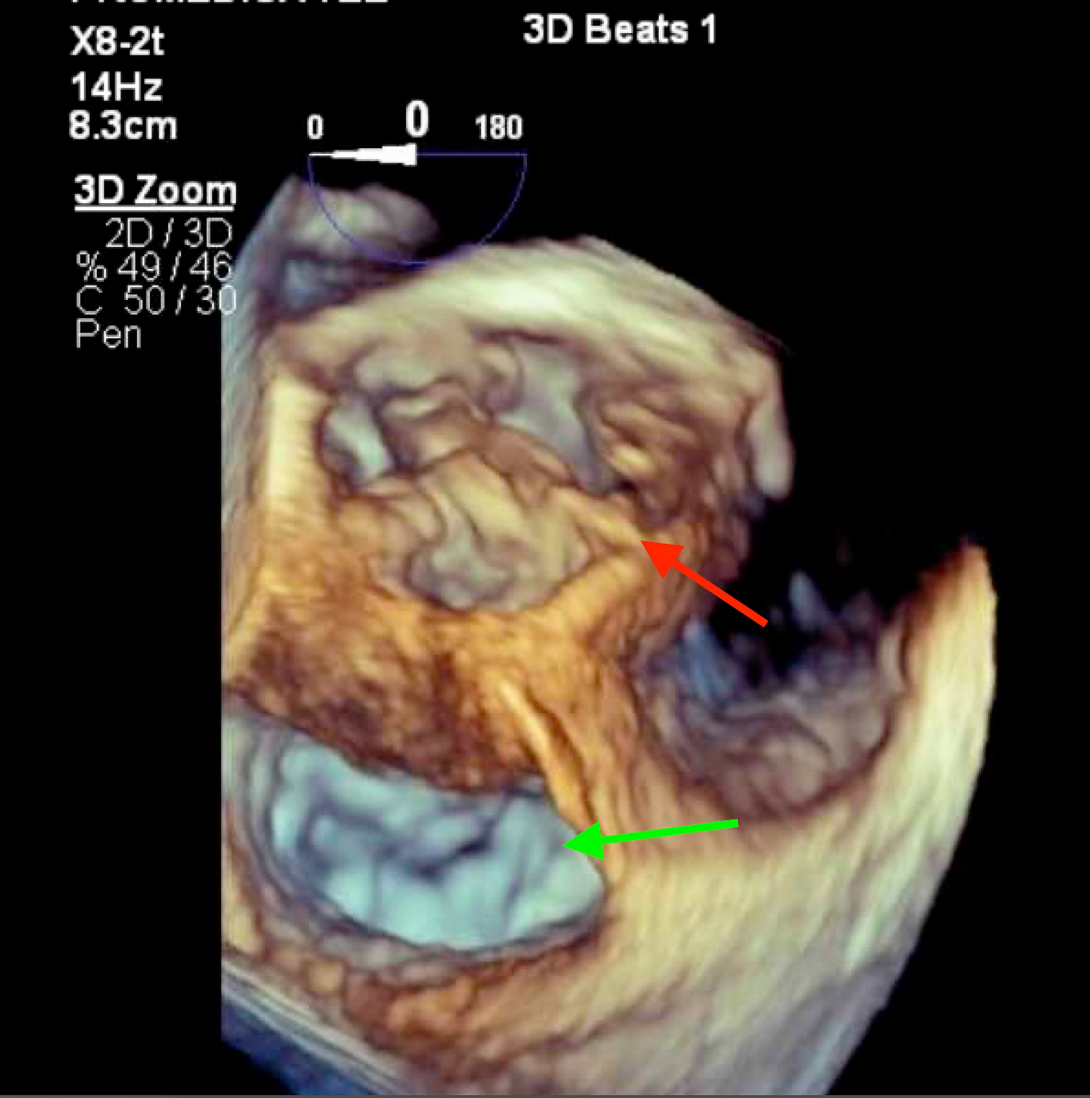
3D recreation on TEE showing vegetations on the aortic valve due to endocarditis and surrounding abscess formation (red arrow), mitral valve seen adjacent (green arrow). TEE, transesophageal echocardiogram

Labs were notable for leukocytosis of 11.1 x 109/uL and troponin of 0.06 ng/mL. Sputum cultures and respiratory pathogen panels were unremarkable. Due to the worrisome findings, a TEE was performed which revealed an aortic valve abscess with vegetations on all three leaflets and an extensive, circumferential periaortic abscess encompassing both coronary ostia and extending towards, but not involving, the tricuspid and mitral valve leaflets. The aortic valve gradient was found to be severely elevated with a mean of 38 mmHg (Figures 3-5).

The patient was transferred to a quaternary care center two days later where a TTE showed possible dehiscence of the prosthetic aortic valve. He was then moved to the ICU and a temporary pacer wire was placed. After another two days, the patient was taken to the OR where he underwent debridement of the aortic root abscess, replacement of the prosthetic aortic valve, and replacement of the aortic root with a homograft. Pathology analysis of the removed valve grew *Enterococcus faecium*. The patient developed complete heart block while recovering in the ICU and received a permanent pacemaker six days after his original operation. Among the three different facilities who cared for this patient until his operation, the patient had 10 blood cultures drawn. All 10 cultures showed no growth.

## Discussion

A study of 233 endocarditis patients found that 10% of patients with IE had a cardiac conduction abnormality and 28% of those with perivalvular extension of infection had conduction abnormalities [4]. Therefore, identifying new onset conduction abnormalities in these patients is a very important diagnostic tool for prompt evaluation of complications and long-term prognosis. New onset arterioventricular (AV) nodal conduction abnormalities highly suspect perivalvular infection as they are relatively specific for this complication compared to uncomplicated IE.

Identifying perivalvular involvement in IE patients is very important because the presence of a perivalvular abscess significantly increases the risk of embolization and mortality [5-6]. The EKG is a cheap and widely available test that may often provide the first hint of perivalvular abscess before a TEE is available. Even if the initial echocardiogram revealed no abscess in a patient with confirmed endocarditis, new rhythm issues should prompt urgent repeat echocardiogram to re-evaluate for extension/abscess. Early recognition and prompt surgical management of this complication will promote better patient outcomes with lower risks for complications.

Lastly, the patient described here received recent antibiotic therapy for a UTI. This case serves as an important reminder that clinicians should remain vigilant for IE even in the absence of positive blood cultures. A detailed history should be elicited from all suspected IE patients with special care taken to review any recent antibiotic therapy. Three sets of blood cultures may fail to show growth in 2%-4% of bacteremia patients and it is important to consider whether previous antibiotic use may increase this chance in a patient [3].

## Conclusions

Early diagnosis of complications arising from IE is imperative in improving patient outcomes. One of the most underrepresented clinical tools to evaluate perivalvular abnormalities remains screening for conduction abnormalities via an EKG. Patients presenting with endocarditis and new conduction issues require urgent echocardiogram imaging to evaluate for perivalvular pathology in a timely manner. Early screening for conduction abnormalities via EKG and subsequently a TEE can allow prompt identification and management of valvular abnormalities and improve patient outcomes. IE is a challenging clinical diagnosis, and Duke’s modified criteria remain a critical tool in guiding clinician thinking. In addition to Duke’s major and minor criteria, clinicians should consider the presence of new conduction abnormalities and recent antibiotic use, especially in patients for whom they have high clinical suspicion of IE despite a “possible” or “rejected” Duke’s criteria score. Prompt identification and management of patients with perivalvular abscess may improve patient mortality.
